# Taxonomic reassessment of *Rhodnius zeledoni* Jurberg, Rocha & Galvão: a morphological and morphometric analysis comparing its taxonomic relationship with *Rhodnius domesticus* Neiva & Pinto

**DOI:** 10.1186/s40850-024-00197-w

**Published:** 2024-03-21

**Authors:** João Paulo Sales Oliveira-Correia, Jader de Oliveira, Hélcio Reinaldo Gil-Santana, Dayse da Silva Rocha, Cleber Galvão

**Affiliations:** 1 Laboratório Nacional e Internacional de Referência em Taxonomia de Triatomíneos, Instituto Oswaldo Cruz, Fiocruz, Rio de Janeiro, Brazil; 2 Laboratório de Entomologia em Saúde Pública, Faculdade de Saúde Pública, Universidade de São Paulo, USP, São Paulo, Brazil; 3 Laboratório de Diptera, Instituto Oswaldo Cruz, Fiocruz, Rio de Janeiro, Brazil

**Keywords:** Chagas disease, Vectors insects, Synonym, Morphological analysis, Geometric morphometry

## Abstract

**Background:**

*Rhodnius zeledoni* was described from a single specimen. Since its description, doubts have arisen regarding the taxonomic status of this species in relation to *Rhodnius domesticus*.

**Methods:**

The present study reviewed and compared *R. zeledoni* with *R. domesticus* based on morphological analysis and head geometric morphometrics.

**Results:**

Our analysis revealed the absence of distinctive diagnostic characters between the two species at specific levels. *Rhodnius zeledoni* and *R. domesticus* show morphological and morphometric similarity, with only minor differences in coloration observed between them. Contrary to previous statements, our analysis showed that *R. zeledoni* and *R. paraensis* are not closely related species, not corroborating previous studies with such an assumption.

**Conclusions:**

Therefore, we formally propose *R. zeledoni* as a junior synonym of *R. domesticus*.

**Supplementary Information:**

The online version contains supplementary material available at 10.1186/s40850-024-00197-w.

## Background

The hematophagous insects of the subfamily Triatominae (Hemiptera, Heteroptera, Reduviidae) are proven or potential vectors of the flagellate protozoan *Trypanosoma cruzi* (Chagas, 1909) (Trypanosomatida, Trypanosomatidae) etiological agent of Chagas disease. Currently, there are 160 species of triatomines, considered as valid, distributed in 18 genera and five tribes [1–3]. The tribe Rhodniini contains two genera, *Rhodnius* Stål, 1859 with 21 species, and *Psammolestes* Bergroth, 1911, with three species, both with species predominantly have arboreal lifestyles. *Rhodnius* species live predominantly in palm trees, and those of the genus *Psammolestes* are strictly associated with nests of different bird species belonging to the families Dendrocolaptidae, Troglodytidae, Furnariidae and Icteridae [4]. While these genera are well characterized and can be easily differentiated from other triatomines, the species belonging to each of them are difficult to differentiate. The main characters distinguishing *Rhodnius* and *Psammolestes* from the other triatomine genera are the apically inserted antennae and distinct callosities behind the eyes [5].

Accordingly, with Neiva and Pinto [6], *Rhodnius domesticus* Neiva & Pinto, 1923 was described based on four males, two from the state of Minas Gerais and two from the state of Rio de Janeiro. They also stated that the “Typo” (Type) would be in the Instituto Oswaldo Cruz, Rio de Janeiro. In the Coleção Entomológica do Instituto Oswaldo Cruz (CEIOC), three type specimens of *R. domesticus* are currently deposited, a female labelled as “Tipo” (Type), and two males labelled as “Cotipos” (Cotypes). The whereabouts of the fourth type specimen are unknown.

*Rhodnius zeledoni* Jurberg, Rocha & Galvão, 2009 was described from a single specimen, found very damaged, in March 2007 in the municipality of Ribeirópolis, Sergipe state, Brazil [7]*.* Jurberg et al. [7] compared *Rhodnius zeledoni* with *Rhodnius paraensis* Sherlock, Guitton & Miles, 1977, from which it differs significantly by the proportions of the head, coloration of the legs and phallic structure of the male genitalia.

Accordingly, with Zhao et al. [8], despite the importance of the study of male genitalia in Reduviidae, among species of *Rhodnius*, most of the descriptions were restricted to the shape of the median process of pygophore. While many species in which more structures were described, the phalli were not everted, resulting in an incomplete documentation of their structure [8]. The latter authors provided a summary of the records of the structures in the species of *Rhodnius*; applied a set of “preferred terms” to the male genitalia of *Rhodnius micki* Zhao, Galvão & Cai, 2021, providing a table defining them, besides listing the previously supposed equivalent terms and literature. Nevertheless, Zhao et al. [8] did not include any observation on the comprehensive and detailed descriptions of the male genitalia of several species of *Rhodnius* by Lent and Jurberg [9], many of them based on completed everted phalli; neither included a comparison of the terminology used by Lent and Jurberg [9] in their comparative table of synonyms of terms. On the other hand, Lent and Jurberg [9] were cited by Zhao et al. [8] to inform that they would have dismissed the diagnostic importance of the female genitalia.

As far as it seems, the list provided by Zhao et al. [8] was not exhaustive, and in some cases, the alleged correspondent structures, as mentioned by other authors, should not be considered as such. For instance, while the alternative term to the median process of pygophore: medial (process of pygophore or pygophore process), has been the one used in comprehensive current papers (e.g. [10, 11]), it was not mentioned in the comparative table of Zhao et al. [8], despite their statement that most of the descriptions of the male genitalia of *Rhodnius* had been restricted to this structure. It is also noteworthy that, possibly because of the difficulty of interpreting the numerous works with descriptions of male genitalia of Triatominae by H. Lent and J. Jurberg, always written in Portuguese, the term “conjunctiva” was misinterpreted. It mostly meant endosoma wall or a part of it in their papers, not the apical part of the intromittent organ, as stated by Zhao et al. [8]. They defined it as “Medial basal sclerite of phallosoma (MBSPh)” a “basal part of a phallosoma, often sclerotized”. However, several structures and terms defined by previous authors and considered by Zhao et al. [8] as being the “MBSPh” were located on median, subdistal or even distal positions of the phallosoma. Therefore, the term must be used just if the sclerite is located at a medial basal position, as defined by its etymology.

In any case, to seek uniformity, when redescribing the male genitalia of *R. domesticus*, we have followed their proposal here as much as possible, except in relation to the abbreviations of the terms, which seemed unnecessary too long. In relation to some structures, such as the median process of endosoma (considered as being the aforementioned “Medial basal sclerite of phallosoma (MBSPh)”) we will maintain the former terminology as used, e.g. by Gil-Santana [12] and Gil-Santana and Oliveira-Correia [13]. Firstly, because of its position, it is not basal but at mid distance between the base and distal portion of phallosoma, or even subdistal. Secondly, because in the species studied here, it is clear that, besides the existence of a subdistal median process of endosoma, other sclerite, that is actually basomedial, will be named accordingly with their proposed terminology (medial basal sclerite of phallosoma). Therefore, it becomes apparent that if both structures are found in the same phallosoma, in different positions and shapes, they cannot be considered the same thing. Thirdly, while the actual median process of endosoma is clearly a structure which lies inside the endosoma, sometimes in need of more clarification or dissection for better visualization, the MBSPh stands out as a projection of the endosoma wall, clearly visible outside of the endosoma, even protruding in relation of it. All of these reasons are evidence that many structures described by other authors and supposed to correspond to MBSPh by Zhao et al. [8] are not the same.

On the other hand, it is noteworthy that the relative position of the apices of the parameres when at rest, i.e., if in contact, close or set apart from each other, although this characteristic may be of taxonomic value (HRG-S pers. obs.), has not been recorded among species of *Rhodnius* so far.

Regarding the male genitalia of *R. domesticus*, Lent [14] and Lent and Wygodzinsky [5] recorded the median process of pygophore as large and subsquared, while Lent [15] additionally provided schematic figures, poor in detail, of ventral and dorsal views of the phallosoma. Lent [15] also emphasized the shape of the median process of pygophore in this species. Lent and Jurberg [9] provided a more comprehensive description of the male genitalia, including more detailed drawings of progressively everted phalli of this species. The description of the male genitalia of *R. zeledoni* was less comprehensive, based on a single specimen, without everting the phallus and less detailed drawings.

However, although *R. domesticus* seems to be the closest species to *R. zeledoni*, no comparisons between them were made by Jurberg et al. [7]. Since its description, the specific validity of *R. zeledoni* remains questionable, the only comparative study was with *R. paraensis*, neglecting the morphological similarity with *R. domesticus*, as highlighted by Galvão [16], Monteiro et al. [17] and Corrêa-do-Nascimento et al. [18]. The objective of this study was to review the taxonomic status of *R. zeledoni* and to compare it with *R. domesticus* and* R. paraensis*. We use morphological and morphometric analyses for the characterization of the species. Additionally, the male genitalia of five specimens of *R. domesticus* were examined and studied to review their morphology, update the terminology of their portions and mainly check if there would be any intraspecific variation of their components.

## Material and methods

### Morphological analyses

Type specimens and non-type specimens from different localities of *R. domesticus*, *R. paraensis* and *R. zeledoni* were examined. The depository institutions, acronyms and respective curators are the following:

- Laboratório Nacional e Internacional de Referência em Taxonomia de Triatomíneos (LNIRTT), Coleção de Triatomíneos do Instituto Oswaldo Cruz (CTIOC), Hugo Lopes Guimarães;

- Laboratório de Biodiversidade Entomológica (LABE), Coleção Entomológica do Instituto Oswaldo Cruz (CEIOC), Márcio Félix;

Both collections are from Fiocruz, Rio de Janeiro, Brazil (see examined material in Additional file 1: Data S1).

**Fig. 1:**
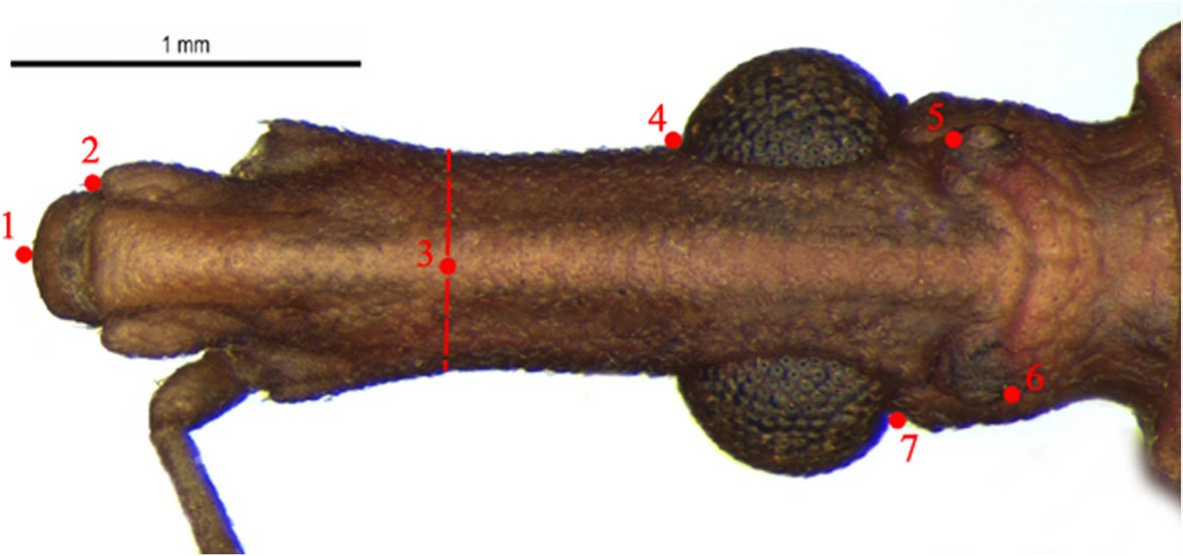
*Rhodnius domesticus*, head, dorsal view of a showing landmarks (target spots) used in morphometric analysis

**Fig. 2:**
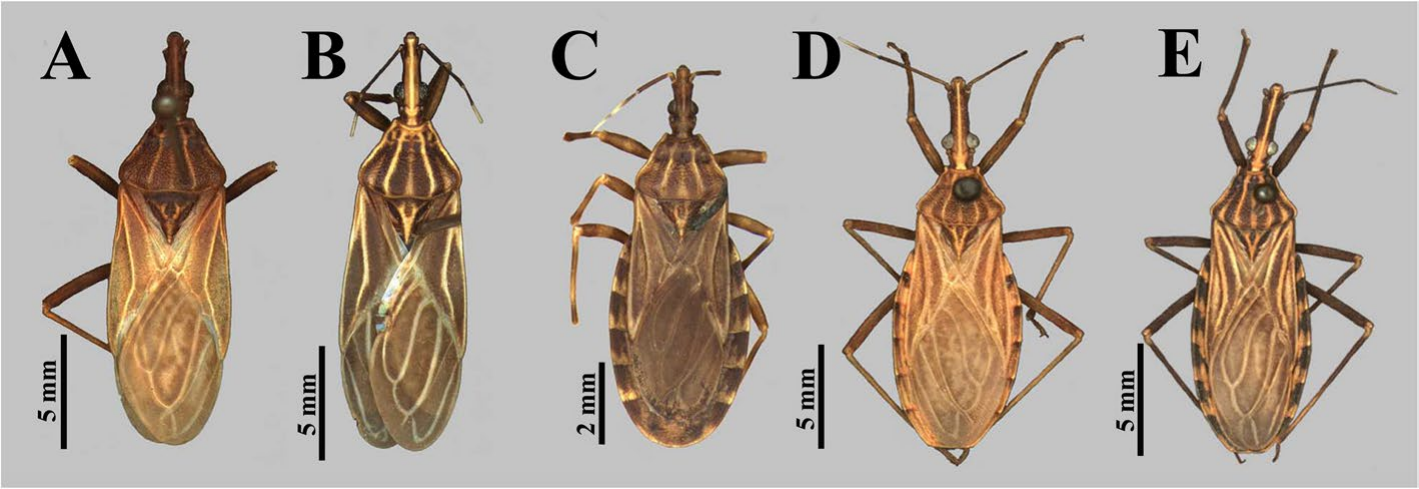
Specimens, dorsal view: **A** *Rhodnius domesticus,* male; **B** *Rhodnius zeledoni*, male holotype; **C** *Rhodnius paraensis*, female holotype; **D** *Rhodnius nasutus*, female; **E** *Rhodnius neglectus*, female

**Fig. 3:**
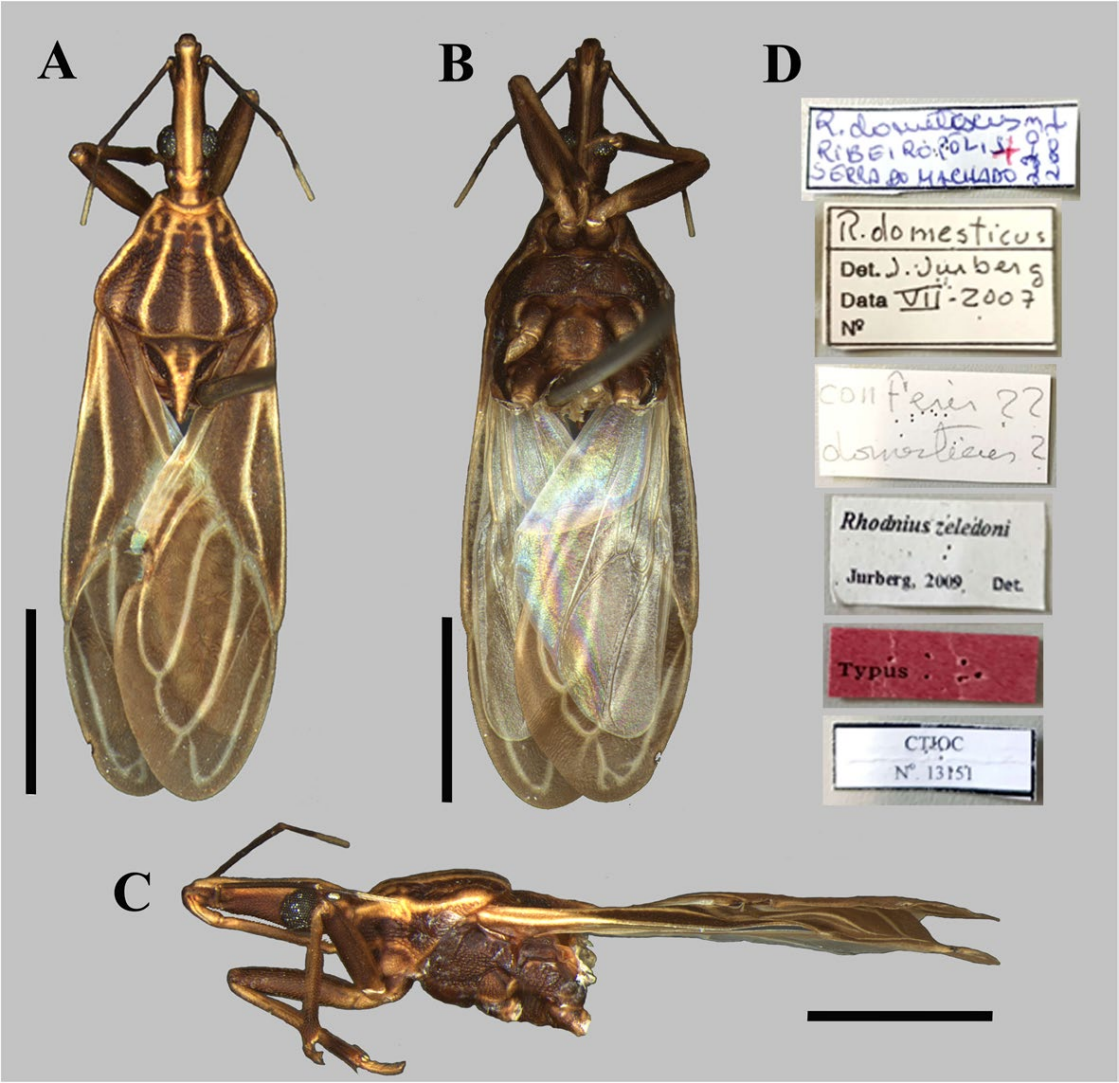
*Rhodnius zeledoni* (male holotype, deposited in CTIOC): **A** Dorsal view; **B** Lateral view; **C** Ventral view; **D** Labels. **A**–**C** Scale 5.0 mm

**Fig. 4:**
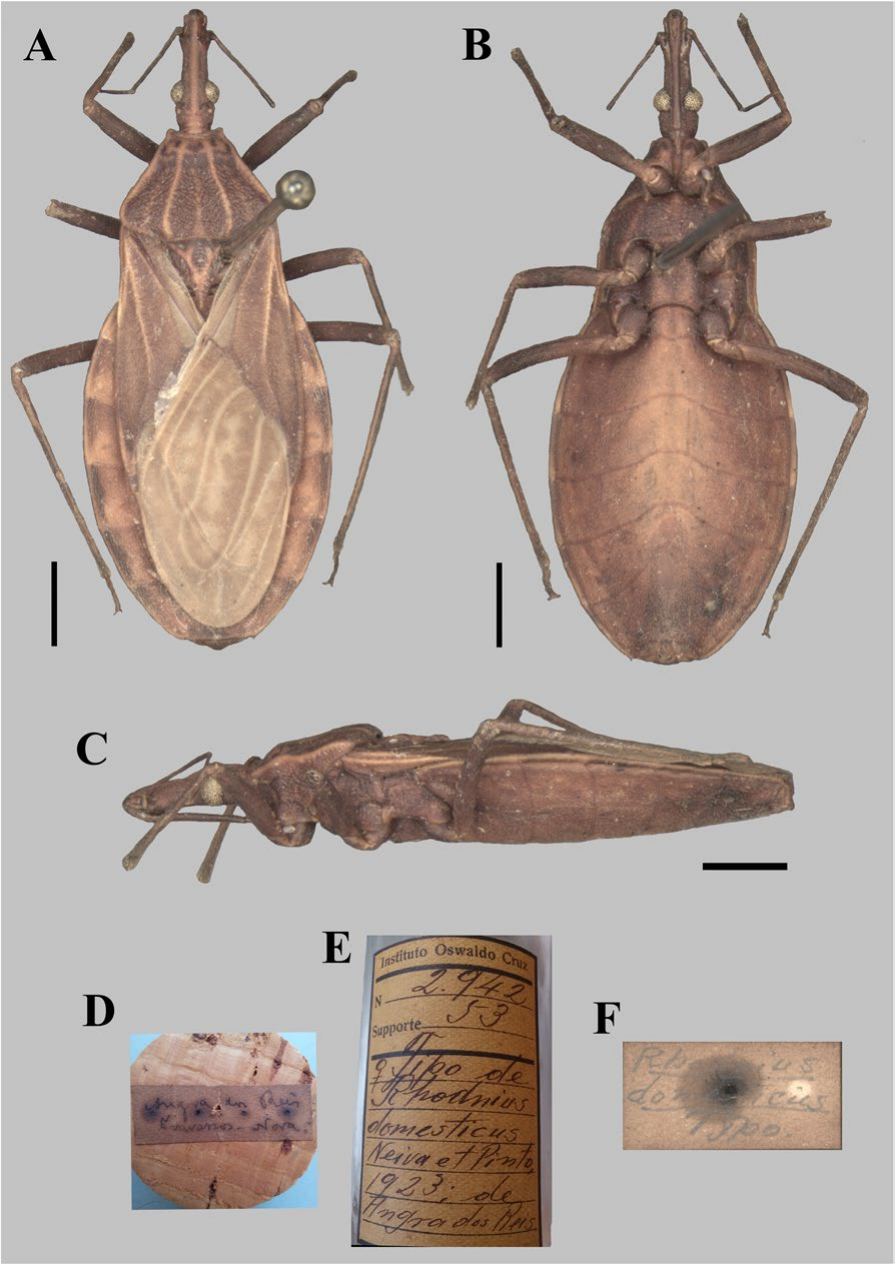
*Rhodnius domesticus* (female holotype, here designated; deposited in CEIOC; originally named as “Tipo” (Type), deposited in CEIOC): **A** Dorsal view; **B** Lateral view; **C** Ventral view; **D** Stopper label; **E** Bottle label; **F** Internal label. **A**–**C** Scale 2.0 mm

**Fig. 5:**
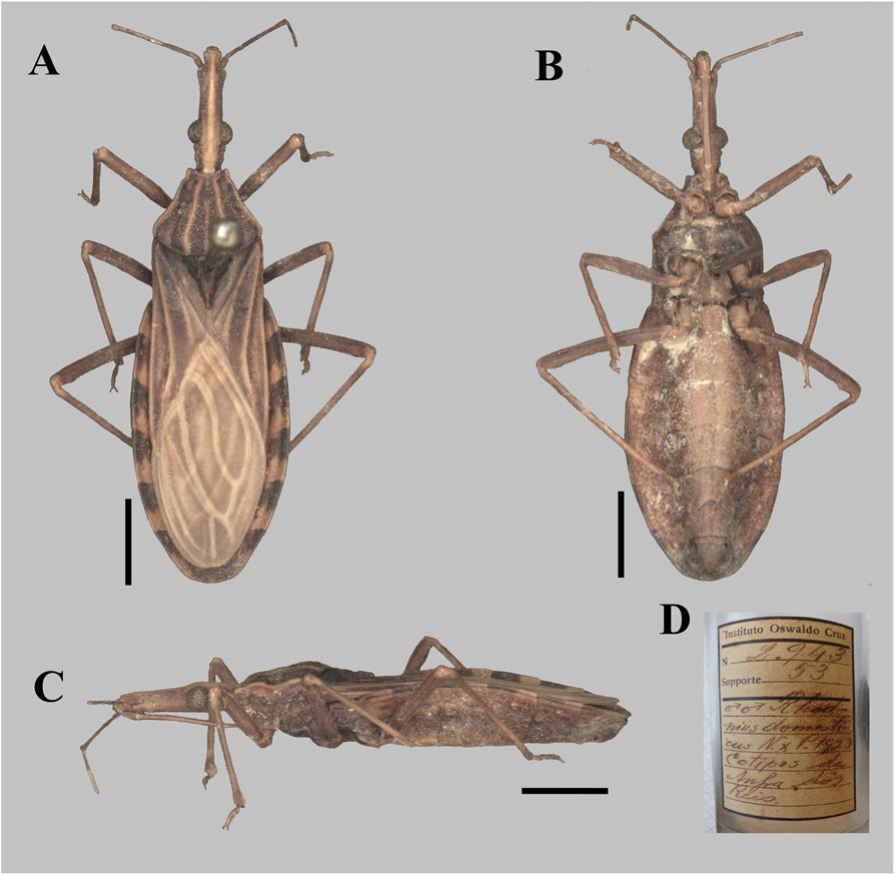
*Rhodnius domesticus*, (male paratype, here designated; deposited in CEIOC, originally named as “Cotipo” (Cotype): **A** Dorsal view; **B** Lateral view; **C** Ventral view; **D** Bottle label. **A**–**C** Scale 2.0 mm

**Fig. 6:**
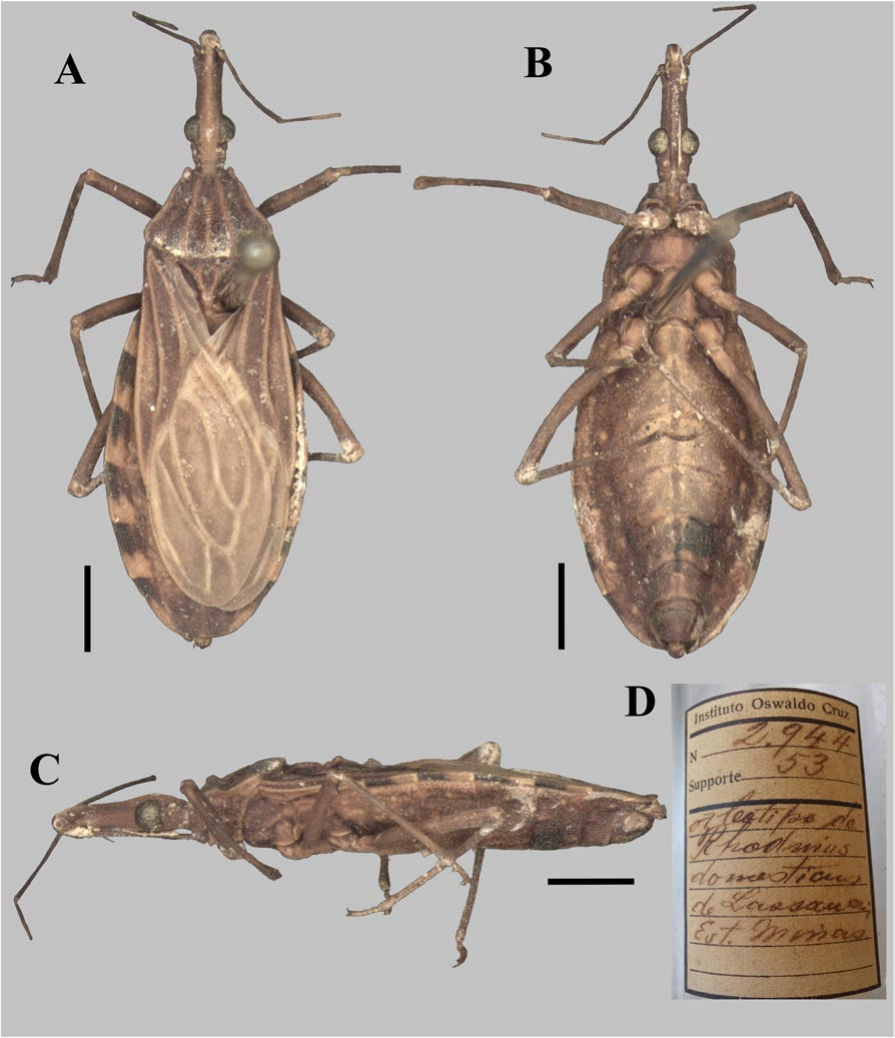
*Rhodnius domesticus* (male paratype, here designated; deposited in CEIOC, originally named as as “Cotipo” (Cotype), deposited in CEIOC): **A** Dorsal view; **B** Lateral view; **C** Ventral view; **D** Bottle label. **A–C** Scale 2.0 mm

**Fig. 7:**
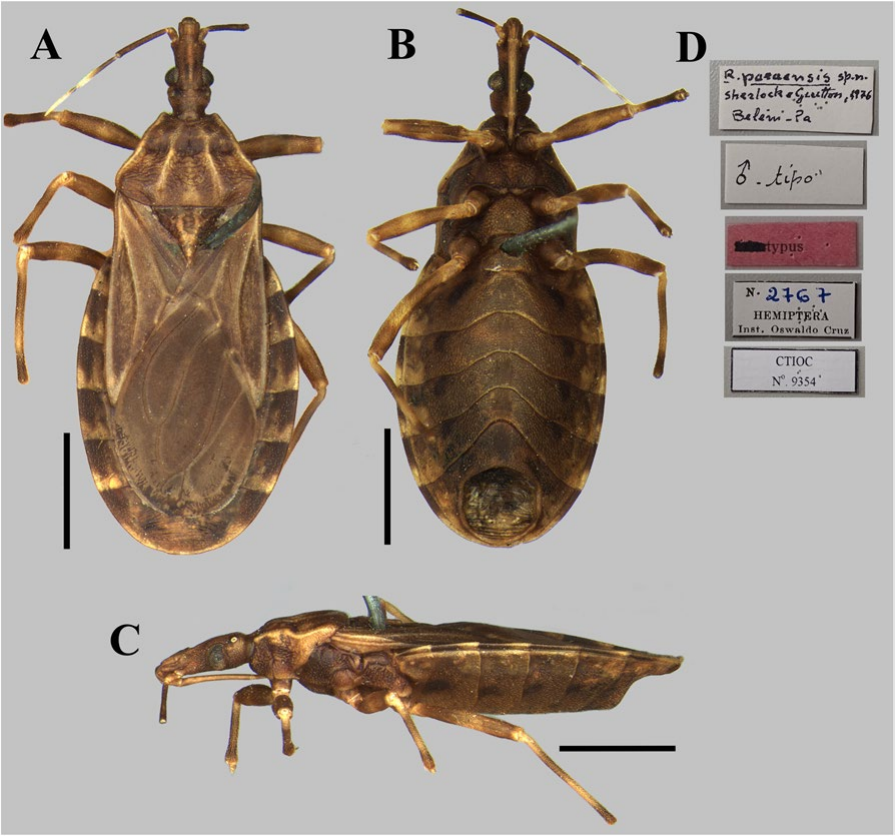
*Rhodnius paraensis* (male holotype, deposited in CTIOC): **A** Dorsal view; **B** Lateral view; **C** Ventral view.; **D** Labels. **A**–**C** Scale 2.0 mm

**Fig. 8:**
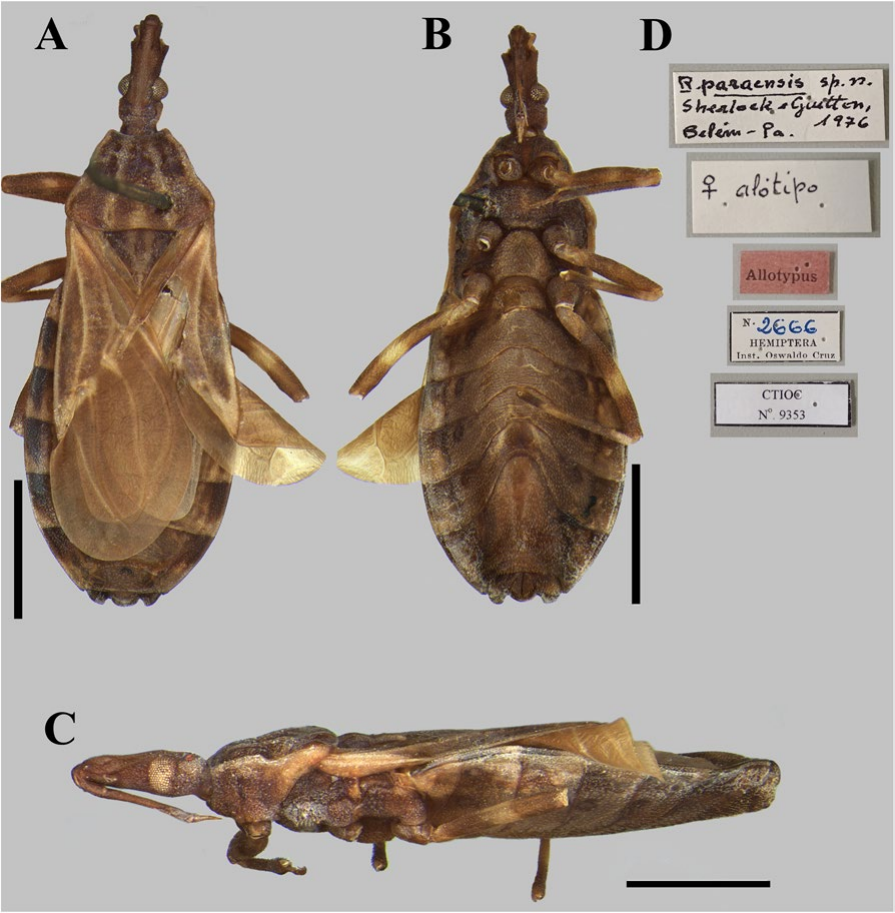
*Rhodnius paraensis* (female “allotypus”, [actually a paratype] deposited in CTIOC): (**A**) Dorsal view; **B** Lateral view; **C** Ventral view; **D** Labels. **A**–**C** Scale 2.0 mm

The photographs, morphological analysis, and measurements of the adults of females and males were made with the Leica DMC 2900 camera attached to the Leica M205C® stereo microscope (Figs. 1, 2, 3, 4, 5, 6, 7, 8 and 10). Images were edited using Adobe Photoshop version 7.0.1. The morphological terminology followed the original species descriptions and Lent & Wygodzinsky [6]. All measurements are in millimeters (mm) (Table 1).

**Table 1:** Morphometric differences between *Rhodnius zeledoni*, *Rhodnius domesticus* and *Rhodnius paraensis*. Scale in millimeters

	*R. zeledoni*	*R. domesticus*	*R. paraensis*
Total length	13^a^	13.31—18	10.5 – 12
Head longer than wide at eye level	1:0.36^a^	1: 0.31- 0.5	1: 0.54 -0.55
Head longer than pronotum	1:0.69^a^	1:0.63–1	1: 0.9–1
Anteocular region longer than postacular region	1:0.32^a^	1: 0,3–0.4	1:0.63–0.64
Maximum width of the head between the eyes	1.11	1.13—1.28	0.87—0.91
Relationship between the width of an eye and the interocular region	1:1.27^a^	1: 1.25–1.5	1: 1.55
Relationship between the visible segments of the labium	1:3.15:0.84^a^	1: 2.8–3.16: 0.8–1	0.9–1:2.6–2.7:0.8–09
Scutellum length in dorsal view	0.67	0.66—0.69	0.7—0.72
Length of scutellar process in dorsal view	0.77	0.76—0.79	0.7—0.72

^a^Retrieved from Jurberg et al.[7]

### Geometric morphometrics analyses

In geometric morphometry analysis, a total of 72 specimens were used. The cephalic capsule was analyzed in 11 specimens of *R. domesticus* (from the Brazilian states of São Paulo, Rio de Janeiro and Santa Catarina), two *R. paraensis* (from the state of Pará) and one *R. zeledoni* (from the state of Sergipe). The external group is formed by species that are also part of the *Rhodnius prolixus* species complex, together with *R. zeledoni* and *R. domesticus*, in addition to occurring in the same region as these species studied, consisting of 27 *Rhodnius nasutus* Stål, 1859 (from states of Ceará, Rio Grande do Norte and Piauí) and 31 *Rhodnius neglectus* Lent, 1954 (from states of Bahia, Goiás, Minas Gerais, Pará and São Paulo) (Fig. 2D, E). The structures were photographed using a Leica DMC 2900 camera attached to a Leica M205C stereomicroscope. The landmark coordinates were registered with TpsDig version v. 2.3.2 (New York, NY, USA) [19] (Table 2; Fig. 1).

**Table 2:** Description of digitized markings on the head

Landmark	Description
1	Anterior border of the anteclypeus in the medial region
2	Apex of the anterior border of the gena
3	Transverse imaginary line cutting through the head at the level of the base of the antennal tubercle, landmark in the median region of the head
4	Anterior border of the eye
5	Anterior border of the ocellus
6	Posterior border of the ocellus
7	Posterior border of the eye

Landmarks were superimposed on Generalized Procrustes Analysis in TPsRelw 6.4 [20–23]. This method allows the calculation of shape variables between taxa after alignment of reference points (to ensure homology), which ensures that all samples are translated around a common origin to remove the effect of position, scaled across the centroid sizes and finally rotated (using the least squares criterion) until the coordinates of the corresponding points align as closely as possible to minimize the effect of orientation differences.

All geometric morphometrics analyses were conducted using MorphoJ [24]. For multivariate analyses, a data matrix consisting of raw data and Procrustes coordinates was employed.

Canonical Variable Analysis (CV) was executed by analyzing the covariance matrix of the Procrustes coordinates. Analysis of Variance (ANOVA) was performed to assess shape variations and deduce differences between species [25]. A factorial map of the first two canonical factors was generated to illustrate the primary results for each species.

The centroid (CS) was derived from the raw coordinate data [26]. Size variables were determined using the isometric estimator, defining the size of the centroid size (CS) [27].

### Analyses of male genitalia

The study of the male terminalia of five non-type specimens of *Rhodnius domesticus* was made by first removing the VIII abdominal segment and pygophore from the abdomen with a pair of forceps (see examined material in Additional file 1: Data S1). Before the following dissections, the male terminalia (segment abdominal VIII and pygophore) were photographed with a Leica DMC 2900 camera attached to the Leica M205C® stereo microscope dried (Fig. 10A, B–D) and after immersion in a liquid solution (Fig. 10C). Then, the genital capsule was immersed in a 20% NaOH solution for 24 h. Following this procedure, a paramere was separated (Fig. 11B), and the phallus was extracted from the pygophore. The phallus was completely everted (Figs. 11C, 12A) by carefully pulling on the dorsal and ventral endosomal walls using a pair of fine forceps. The dissected structures (Figs. 11, 12 and 13) were studied and photographed in glycerol using a digital camera (Sony DSC-W830). Images were edited using Adobe Photoshop CS6. The terminology applied to the male genital characteristics mainly follows Zhao et al. [8] but also Lent and Jurberg [9], Lent and Wygodzinsky [5], Jurberg et al. [7], Gil-Santana [12] and Gil-Santana and Oliveira-Correia [13].

## Results

### Morphological characterization and geometric morphometry

The only difference found between *R. zeledoni* and *R. domesticus* was in the tonality of the general coloration, with *R. zeledoni* light brown and *R. domesticus* orange brown (Figs. 2A**-**B; 3, 4 and 5; Table 1).

**Fig. 9:**
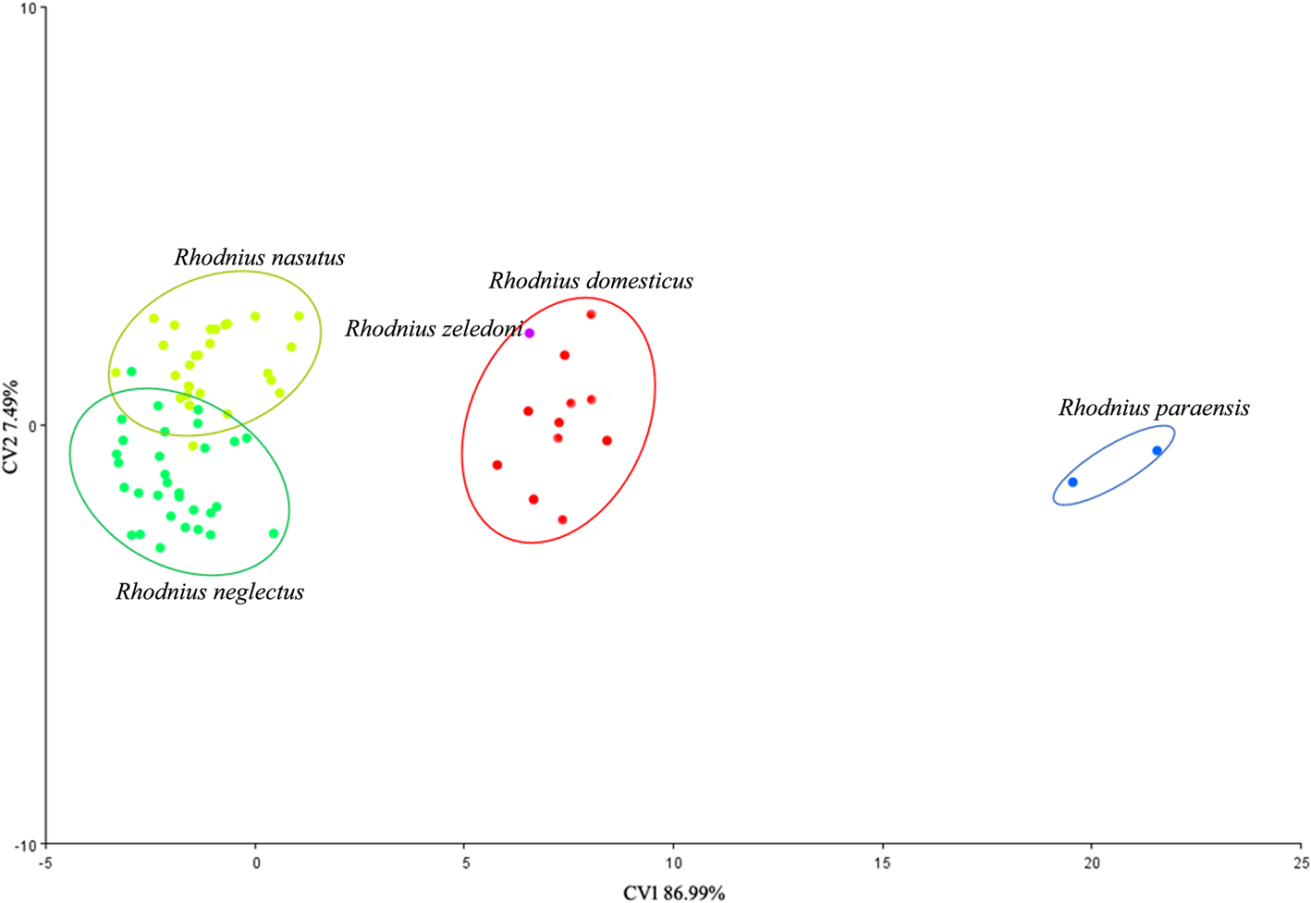
Factorial map of the four species studied in the space of canonical axes (CVA1 and CVA2) resulting from the canonical analysis carried out using Procrustes coordinates. The ellipses represent the projection of each species. The purple circle represents *Rhodnius zeledoni*

The CS ANOVA is insignificant for head size, with p < 0.0003. For the conformation of the head, it was possible to observe the projection of the four species defined by the canonical axes CV1 and CV2, describing the differences between the taxa, with values 86.99% and 7.49%, respectively (Fig. 9). The elliptical projections show the organizational profiles of the species, being that the type specimen originally designated as *R. zeledoni* is not distinct from specimens of *R. domesticus* (Fig. 9).

Procrustes distance provides strong evidence that *R. zeledoni* and *R. domesticus* are the same species (Table 3). Pairwise comparisons revealed that the two species differ by 0.032. Comparing *R. zeledoni* with the other species, we will have the following values: *R. paraensis* (0.106); *R. nasutus* (0.083); and *R. neglectus* (0.086).

**Table 3:** Procrustes distance among species

	***R. domesticus***	***R. nasutus***	***R. neglectus***	***R. paraensis***
*Rhodnius nasutus*	0.066			
*R. neglectus*	0.671	0.051		
*R. paraensis*	0.117	0.18	0.178	
*R. zeledoni*	0.032	0.083	0.086	0.106

The examined specimens of *R. paraensis* and *R. zeledoni* showed the following diagnostic characters: ratio between head length and pronotum; maximum pronotum width; ratio of head length to width at eye level; proportion between the anteocular and postocular region; proportion between the visible segments of the labium; and coloring and chromatic pattern of the antennae and legs (Figs. 2B, C; 7 and 8; Table 1). It was impossible to analyze and compare the characters of the abdomen between the two species because this portion was found to be currently absent in the holotype of *R. zeledoni*.

### Description of male genitalia structures

**Fig. 10:**
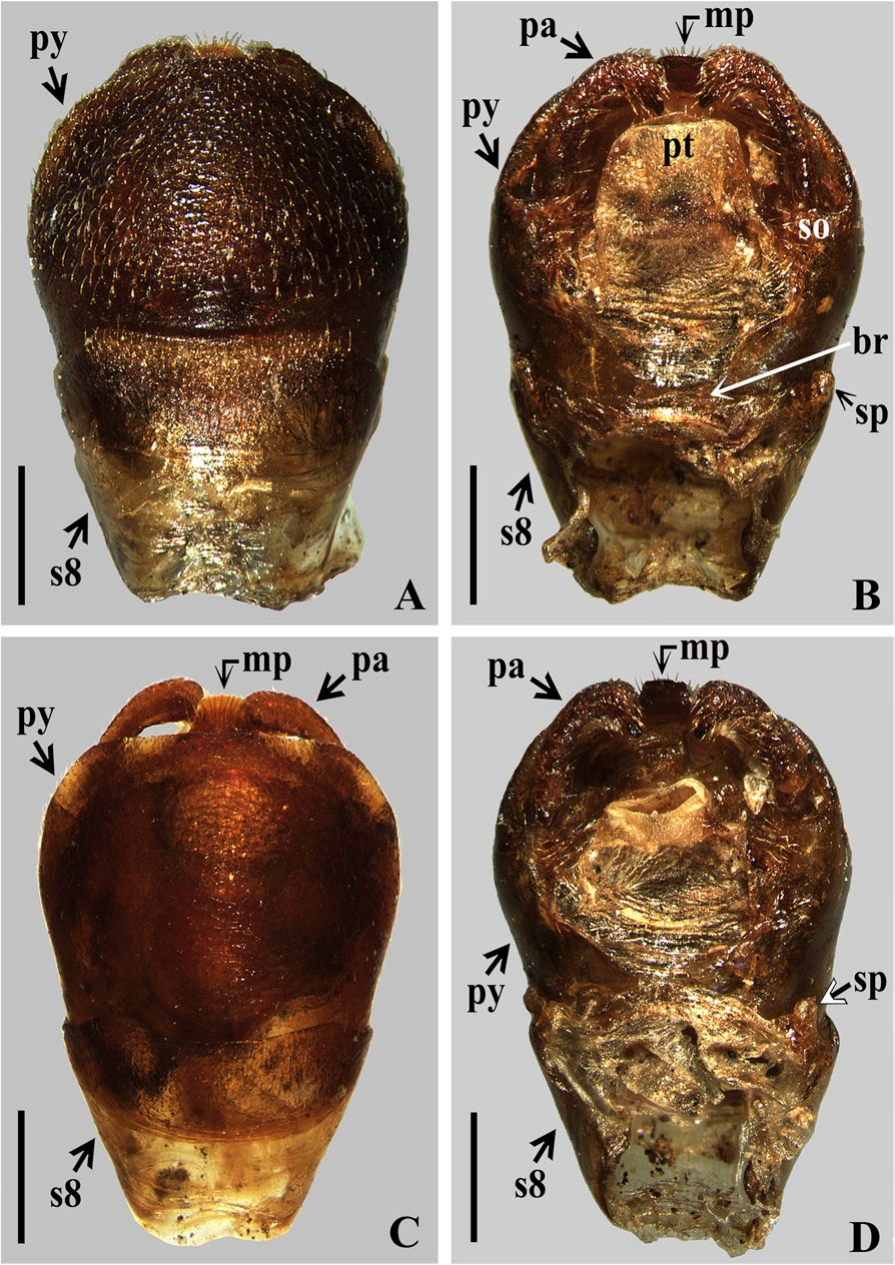
*Rhodnius domesticus*, male. **A**–**D**, abdominal segment VIII and pygophore, scale bar 0.5 mm; **A** and **C**, ventral view, **C**, immersed in liquid solution; **B** and **D**, dorsal view; br: transverse bridge; pa: paramere; mp: median process of pygophore; py: pygophore; s8: abdominal segment VIII; so: socket of insertion of paramere; sp:spiracle

Male Terminalia (Fig. 10): abdominal segment VIII (s8): ventral portion: sclerotized, becoming wider towards posterior margin; both basal and distal margins curved, the former more than the latter; distal portion of ventral portion with scattered thin, short to slightly elongated setae; dorsal portion membranous and narrower; spiracles (sp) on dorsal margin of ventral portion (Fig. 10 B, D).

**Fig. 11:**
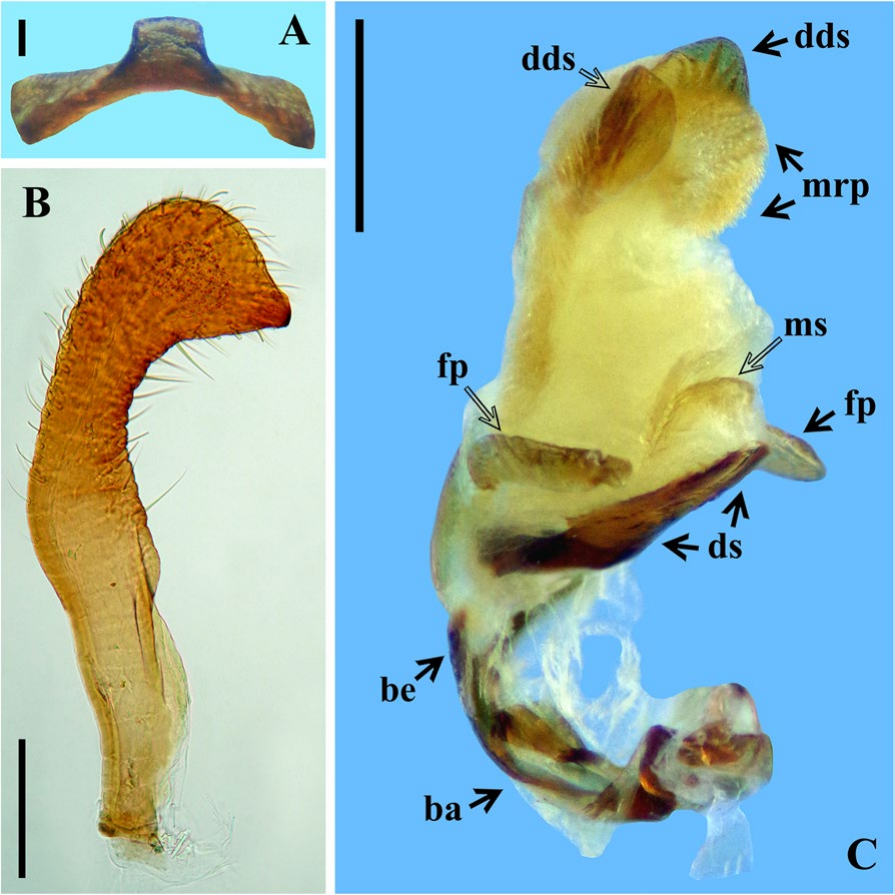
*Rhodnius domesticus*, male genitalia. **A**, median process of pygophore, dorsal view, scale bar 0.1 mm; **B**, right paramere, lateral view, scale bar 0.2 mm; **C**, extended phallus, lateral view, scale bar 0.5 mm, dorsal view; ba: basal plate arms; be: basal plate extension; ds: dorsal phallothecal sclerite; dds: distal dorsal sclerites of endosoma; fp: flap-like prolongations of phallosoma; mrp: median rounded protuberance; ms: medial basal sclerite of phallosoma

**Fig. 12:**
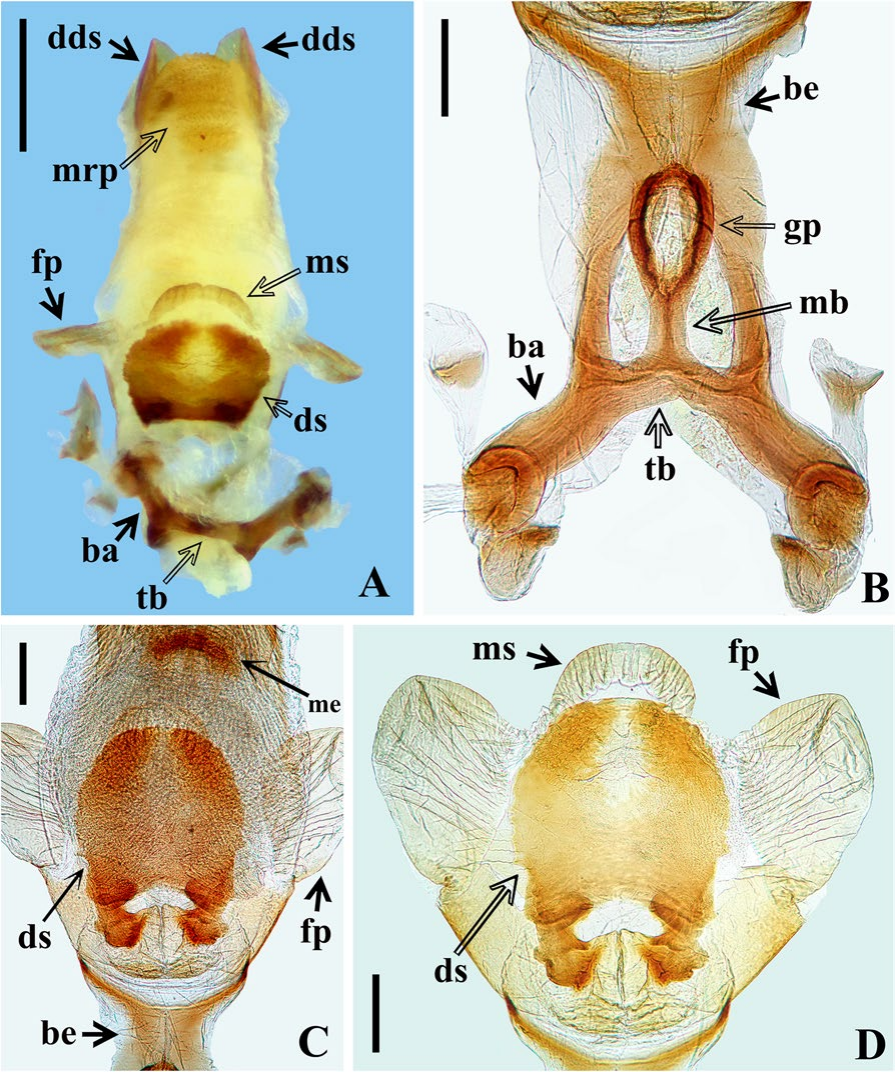
*Rhodnius domesticus*, male genitalia, dorsal view. A, extended phallus, scale bar 0.5 mm; **A**–**D**, scale bar 0.2 mm; **B**, articulatory apparattus; **C**–**D**, phallosoma, **C**, basal portion; **D**, central portion; ba: basal plate arms; be: basal plate extension; ds: dorsal phallothecal sclerite; dds: distal dorsal sclerites of endosoma; fp: flap-like prolongations of phallosoma; gp: gonopore process; mb: median bridge; me: median process of endosoma; mrp: median rounded protuberance; ms: medial basal sclerite of phallosoma tb: transverse bridge of basal plate

**Fig. 13:**
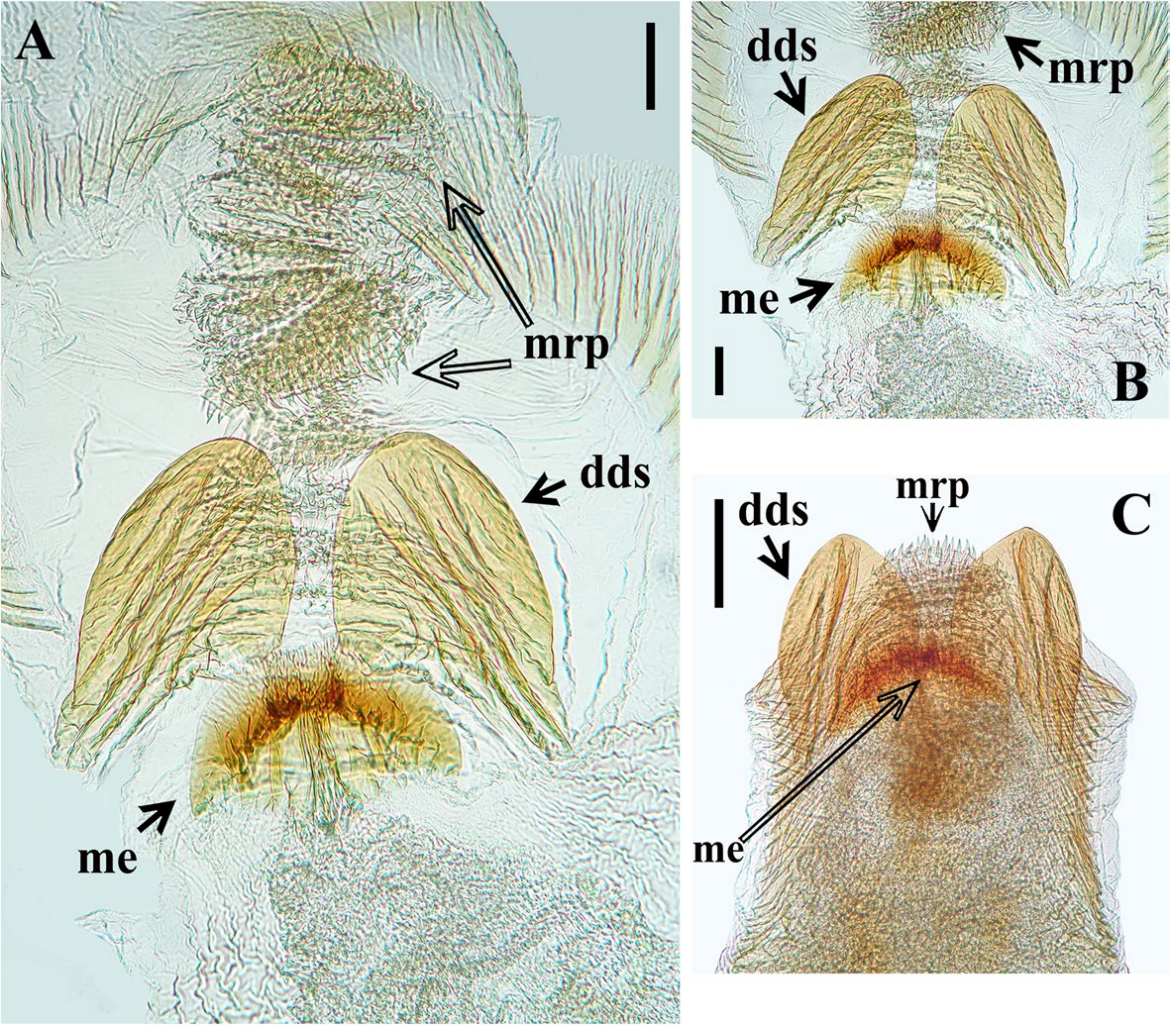
*Rhodnius domesticus*, phallosoma of male genitalia, dorsal view. **A**–**B**, middistal portion, scale bar 0.1 mm; C, distal portion, scale bar 0.2 mm; dds: distal dorsal sclerites of endosoma; me: median process of endosoma; mrp: median rounded protuberance

Male genitalia (Figs. 10, 11, 12 and 13): pygophore (py) with numerous short, curved, thin, decumbent, pale setae on its exposed surface, ventrally (Fig. 10A); subrounded in ventral and dorsal views (Fig. 10A–D); in dorsal view (Fig. 10B, D): between anterior and posterior genital openings, a moderately broad transverse bridge (br); socket of insertion of paramere (so) approximately in basal portion of distal third of pygophore, and with numerous, somewhat long, erect setae inserted above it, medially; posterior genital opening covered by a smooth membrane; proctiger (pt) large, subsquared. Parameres (pa) symmetrical, moderately elongated, their apices far when in resting position, beside or shortly above median process of pygophore (mp) in dorsal view; with short protuberances at submedian proximal portions; strongly curved at apical third, enlarged apically with an acute and sclerotized tooth at its tip; integument smooth and rugous on approximately basal and distal halves, respectively; glabrous on approximately basal half and covered by short, curved and numerous setae on outer and lateral surfaces and less numerous, straight, thin and longer setae on internal surface of distal half. Median process of pygophore (mp) sclerotized, visible on ventral and dorsal views of pygophore, subsquared in shape (Figs. 10B–D, 11A).

Phallus (Figs 11C, 12 and 13): articulatory apparatus moderately bent dorsally when at rest in relation to the phallosoma (Fig. 11C); when extended, in dorsal view (Fig. 12B): basal plate arms (ba) moderately elongated; narrower just above transverse bridge of basal plate (tb), the latter and basal plate arms (ba) forming a subrectangular set; transverse bridge of basal plate (tb) somewhat curved at middle portion, where it meets the median bridge (mb) which ends at the large and somewhat sclerotized gonopore process (gp); basal plate extension (be) short (Fig. 12 B–C). Dorsal phallothecal sclerite (ds) sclerotized, with a pair of bulbous protuberances basally and mostly formed by a rounded flat plate faintly sclerotized, even less at midportion of distal half, apical margin rounded (Figs. 11C, 12A, C–D).

Endosoma wall mostly rugous, with ill-defined striations; smooth and somewhat sclerotized basally; at level of the plate of dorsal phallothecal sclerite, with a pair of large lateral flap-like prolongations of phallosoma (fp), not sclerotized, with several linear grooves along it (Figs. 11C, 12 A, C, D); just above the dorsal phallothecal sclerite, at median portion, with a short medial basal sclerite of phallosoma (ms), narrower than the dorsal phallothecal sclerite and with several longitudinal parallel linear grooves (Figs. 11C, 12 A, D); at distal portion the endosoma wall forms a somewhat large dorsal median rounded protuberance (mrp) covered by numerous acute small processes (Figs. 11C, 12A, 13A–C). Endosoma processes (Figs. 12C, 13A–C): a flat median process of endosoma (me) at subapical portion, subhemisphaerical, sligthly sclerotized, better visualized after dissection of the endosoma, lying just below the pair of the distal dorsal sclerites of endosoma (dds); the latter flat, sclerotized, with linear parallel grooves, somewhat elongated, lying latero-distally in resting position; when dissecting the endosoma it is possible to observe that these sclerites and the median process of the endosoma (me) are connected by fibrous connections.

## Discussion

In relation to the type specimens of *R. domesticus* (Figs. 4, 5 and 6), although Neiva and Pinto [6] stated that its description was based on four males, at least one type specimen is a female (Fig. 4), which is by coincidence the species which they labelled as “Tipo” (Type) of the new species. On the other hand, there are some issues that must be updated under the scope of the International Code of Zoological Nomenclature (ICZN) [28]. Firstly, “if an author when establishing a new nominal species-group taxon states in the original publication that one specimen, and only one, is the holotype, or "the type", or uses some equivalent expression, that specimen is the holotype fixed by original designation”. (ICZN, Art. 73.1.1, the highlighted words by us). Therefore, the “Tipo” (Type) (Fig. 4) must clearly be considered as the holotype of *R. domesticus* and labeled as such. Secondly, considering that “an author should not use the term "cotype", e.g. in the sense of syntype or paratype.” (ICZN, Recommendation 73E), and that** “**after the holotype has been labelled, any remaining specimens of the type series [Art. 72.4.5] should be labelled "paratype" to identify the components of the original type series.” (ICZN, Recommendation 73D), the other extant type-specimens (Figs. 5–6) must be considered paratypes. The fourth type specimen mentioned by Neiva and Pinto [6], if found in the future, must be considered as a paratype as well.

It is noteworthy that when describing *Rhodnius paraensis*, Sherlock et al. [29] stated that the type specimens deposited in CTIOC were the male holotype and a female paratype (Figs. 7, 8). Regarding the female paratype, although the specimen has a label designating it as “allotypus” (allotype) (Fig. 8), it must be considered only as a paratype following the statement of the publication of Sherlock et al. [29] and because the ICZN does not regulate this term and recommend that it must be considered as a paratype (Recommendation 72 A).

In March 2007, the technical team of the “Laboratório Central de Saúde Pública de Sergipe” (HEMOLACEN/SE) collected a dry specimen of a specimen identified as *Rhodnius domesticus* in the municipality of Ribeirópolis, Sergipe State, Brazil. Due to the poor condition of the specimen, it was sent to LNIRTT, Instituto Oswaldo Cruz, Rio de Janeiro, for diagnosis confirmation. The insect was promptly confirmed as *R. domesticus* by the team of LNIRTT (C Galvão and DS Rocha), and a confirmation report was sent to HEMOLACEN (April 2007). Posteriorly, the same specimen was confirmed as *R. domesticus* by J Jurberg (July 2007) (Fig. 2D). Surprisingly, the specimen was reclassified as a new species by the first author (J Jurberg), the respective manuscript submitted for publication in December 2007, and published in March 2009, without revision by the last authors. The original description appears on a very poor paper, with some undiagnostic drawings and without a single photograph of the specimen. The only type specimen of *R. zeledoni* (male holotype) was deposited in the Coleção Herman Lent, CTIOC, at the Instituto Oswaldo Cruz. However, after publication, the holotype of *R. zeledoni* remained missing for several years. After an exhaustive search in the CTIOC, the specimen was found in a different drawer, making it possible to carry out the present paper. Notably, the label “n° 3078”, which would be the respective number of the specimen in the CTIOC, was not found. Therefore, the identification of the holotype was only possible by the confirmation of C. Galvão, who recognized the specimen as such.

Jurberg et al. [7] considered *R. zeledoni* similar to *R. paraensis*, but our morphological and morphometric review made it clear that *R. zeledoni* is not close to that species. Furthermore, the procrutes distance analysis revealed *R. nasutus* and *R. neglectus* close to *R. zeledoni* and *R. domesticus* and, in turn, more distant from *R. paraensis*, corroborating the already established hypothesis of the formation of the *R. prolixus* species complex.

This assumption is based on the thorough knowledge of the morphological characteristics of these two species [5–7]. On the other hand, our analyses found that *R. zeledoni* and *R. domesticus* have the same size, and proportions between the morphological structures of the head and male genitalia. It is noteworthy that the variation in the coloration of the body between the two species can be recognized as a phenotypic variation between the populations resulting from ecological diversity.

At first glance, regarding the recent contribution by Zhao et al. [3], their suggestion of “preferred terms” to several structures, mainly those of male genitalia of reduviids, may be considered a good initiative. However, unfortunately, because they missed important previous references and considered different structures as being the same, it is necessary to be careful about each of their “preferred term”. Therefore, a future, more comprehensive proposal, based not only on previous literature but also committed to searching for homologies to name the structures in different taxa, would be very useful to uniformize the terminology of the male genitalia in Reduviidae.

The examination of five male genitalia of *Rhodnius domesticus* reveals only a slight variation in the position of the apices of parameres, which lie beside or shortly above median process of pygophore (mp) in dorsal view. On the other hand, the other structures did not reveal variation, while they seemed very similar to the description provided by Lent and Jurberg [9]. Yet, although the description of the male genitalia of *R. zeledoni* by Jurberg et al. [7] had been less comprehensive, it seems compatible with that of *R. domesticus*. Because they did not evert the phallus, nor made any dissection of the endosoma, it is impossible to compare all structures. Even so, judging by their description and figures, the comparable structures of the male genitalia such as: pygophore, median process of pygophore, parameres, articulatory apparatus, phallothecal sclerite, lateral flap-like prolongations of phallosoma and the pair of the distal dorsal sclerites of endosoma, show a high level of similarities between *R. domesticus* and *R. zeledoni*, reinforcing the synonym proposed here based in other characteristics.

There is no information about the geographic distribution, biology and ecology of the specimen described as *R. zeledoni,* however the municipality where the specimen was found agrees with the geographical distribution of *R. domesticus* as demonstrated by Galvão & Gurgel-Gonçalves [16], Corrêa-do-Nascimento et al. [30] and Corrêa-do-Nascimento & Leite [18]. According to the last authors, after reviewing and analyzing information on the geographic distribution of *R. domesticus*, and creating robust ecological niche models, they found that Ribeirópolis is a geographic area with adequate conditions for the occurrence of *R. domesticus*. Thus, the authors also believe that *R. zeledoni* is synonymous with *R. domesticus*, corroborating our hypothesis. We emphasize the importance of depositing and preserving specimens and cataloging information clearly in scientific collections, which are fundamental to solving taxonomic problems, as observed in *R. zeledoni*.

## Conclusion

### Taxonomy

Order Hemiptera.

Suborder Heteroptera.

Family Reduviidae.

Subfamily Triatominae.

Tribe Rhodninii.

Genus *Rhodnius* Stål, 1859.

*Rhodnius domesticus* Neiva & Pinto, 1923.

*Rhodnius zeledoni* Jurberg, Rocha & Galvão, 2009, syn. nov.

### Supplementary Information

**Additional file 1.** **Data S1.** Examined material of the studied species.

## Data Availability

All relevant data are within the paper.
