# Supplementary material for: Taxonomic reassessment of Rhodnius zeledoni Jurberg, Rocha & Galvão: a morphological and morphometric analysis comparing its taxonomic relationship with Rhodnius domesticus Neiva & Pinto
Source: BMC Zool. 2024 Mar 21;9:6. doi: 10.1186/s40850-024-00197-w (PMC10956182; doi:10.1186/s40850-024-00197-w)
Supplement: Supplementary file 1 — Additional file 1. Data S1. Examined material of the studied species. [file 40850_2024_197_MOESM1_ESM.docx]

**Supplementary material**

**Data S1**. Examined material of the studied species

The boldface text represents specimens used for geometric morphometric analysis and blue text represent specimen used for analyzed male genitalia. The boldface text represents specimens used for geometric morphometric analysis. A slash (/), separates the lines, a double slash (//) different labels and a semicolon (;) separates the species. The material is listed as per the original specimen label.

*Rhodnius zeledoni*

**Type material. *R. domesticus* / Ribeirópolis / Serra do Machado / 22-03 / 2007 //Conferir?? / *domesticus*? // *R. domesticus /* Det. J. Jurberg / Data VII – 2007 / Nº // *Rhodnius zeledoni* / Jurberg, 2009 Det. / CTIOC / Nº 13151 / Typus ♂.**

*Rhodnius paraensis*

**Type material. *R. paraensis* sp. n / Sherlock & Guitton, 1976 / Belém-PA // ♀ alótipo // N 2666/ Hemiptera / Inst. Oswaldo Cruz // Allotypus // CTIOC/ Nº 9353; *R. paraensis* sp. n / Sherlock & Guitton, 1976 / Belém - PA // ♂. Tipo // Typus // N. 2767 / Hemiptera / Inst. Oswaldo Cruz // CTIOC / N° 9354.**

*Rhodnius domesticus*

**Type material**. Female Holotype (here designated): [label on the glass:] Instituto Oswaldo Cruz/ N 2.942/ Supporte 53/ ♀ tipo de *Rhodnius / domesticus/* Neiva e Pinto/ 1923 de / Angra dos Reis // [label on bottle roll:] Angra dos Reis / Travassos– Nova // [label of the specimen:] *Rhodnius / domesticus*/ Typo; Male Paratype (here designated): [label on the glass:] Instituto Oswaldo Cruz/ N 2.943/ Supporte 53/ ♂♂ *Rhod- / nius domesti- /cus /* N x P. 1923 / cotipos de / Angra dos / Reis; Male Paratype (here designated): [label on the glass:] Instituto Oswaldo Cruz/ N 2.944 / Supporte 53/ ♂ Cotipo de /*Rhodnius* / *domesticus /*de Lassance “;”/ Est. Minas. (CEIOC)

**Non-type material**. **Angra dos Reis / Est. Do Rio / Brasil / Travassos col. // N. 789 / Hemiptera / Inst. Oswaldo Cruz // Paratype // CTIOC / N° 11637; *Rhodnius domesticus* / Neiva & Pinto //Brasil/ São Paulo/ Pariquera Açu / VI-74 // 226 // Coleção / Rodolfo Carcavallo // CTIOC / N° 3426 ♀; Brasil / São Paulo / Pariquera Açu / VI-74 // 228 // Coleção / Rodolfo Carcavallo // CTIOC / Nº 7600 ♀; *Rhodnius* do/ mesticus / Neiva & Pinto / R. Carcavallo det. 1973 // Brasil / São Paulo / Iguapé / VI-84 // 224 // Coleção/ Rodolfo Carcavallo // CTIOC / N° 7601 ♀; Brasil/ São Paulo/ Pariquera Açu / VI-74 // 236 // Coleção / Rodolfo Carcavallo // CTIOC / N° 7602 ♀; *Rhodnius domesticus* / Neiva e Pinto, 1923 / 3-947 // Brasil – São Paulo, / Juquiá, Faz. Poço Grande / 6-9.IV.1940 – F. Lane & / Trav. Fo. & C. Carcavallo // N. 651 / Hemiptera / Inst. Oswaldo Cruz // CTIOC / Nº 11936;** ***Rhodnius domesticus //* Florianópolis – Santa Catarina // Col. Aldo Valente// 139// CTIOC/ N° 14429 ♂; *Rhodnius domesticus //* Florianópolis – Santa Catarina // Col. Aldo Valente// 139// CTIOC/ N° 14430 ♂; *Rhodnius domesticus //* Florianópolis – Santa Catarina // Col. Aldo Valente// 139// CTIOC/ N° 14431 ♂; *Rhodnius domesticus //* Florianópolis – Santa Catarina // Col. Aldo Valente// 139// CTIOC/ N° 14432 ♂; *Rhodnius domesticus //* Florianópolis – Santa Catarina // Col. Aldo Valente// 139// CTIOC/ N° 14433 ♂.**

*Rhodnius nasutus*

**Non-type material. Brasil / R. G. do N / Lab 83 // Coleção Rodolfo Carcavallo // 272 // CTIOC / Nº 7507 ♂; Brasil / R. G. do N / Lab 83 // Coleção Rodolfo Carcavallo // 273 // CTIOC / Nº 7508 ♂; Brasil / R. G. do N / Lab 83 // Coleção Rodolfo Carcavallo // 275 // CTIOC / Nº 7509 ♂; Brasil / R. G. do N / Lab 83 // Coleção Rodolfo Carcavallo // 276 // CTIOC / Nº 7510 ♂; Brasil / R. G. do N / Lab 83 // Coleção Rodolfo Carcavallo // 277 // CTIOC / Nº 7511 ♂; Brasil / R. G. do N / Lab 83 // Coleção Rodolfo Carcavallo // 278 // CTIOC / Nº 7512 ♀; Brasil / R. G. do N / Lab 83 // Coleção Rodolfo Carcavallo // 280 // CTIOC / Nº 7514 ♂; Brasil / R. G. do N / Lab 83 // Coleção Rodolfo Carcavallo // 282 // CTIOC / Nº 7515 ♂; Brasil / R. G. do N / Lab 83 // Coleção Rodolfo Carcavallo // 284 // CTIOC / Nº 7517 ♂; Brasil / R. G. do N / Lab 83 // Coleção Rodolfo Carcavallo // 285 // CTIOC / Nº 7518 ♂; Brasil / R. G. do N / Lab 83 // Coleção Rodolfo Carcavallo // 286 // CTIOC / Nº 7519 ♂; Brasil / R. G. do N / Lab 83 // Coleção Rodolfo Carcavallo // 290 // CTIOC / Nº 7523 ♀; Brasil / R. G. do N / Lab 83 // Coleção Rodolfo Carcavallo // 292 // CTIOC / Nº 7525 ♂; Brasil / R. G. do N / Lab 83 // Coleção Rodolfo Carcavallo // 293 // CTIOC / Nº 7526 ♂; Brasil / R. G. do N / Lab 83 // Coleção Rodolfo Carcavallo // 294 // CTIOC / Nº 7527 ♂; *Rhodnius* / *nasutus* / R. Carcavallo det. 1984 // Brasil / R. G. do N / Lab 83 // Coleção Rodolfo Carcavallo // 295 // CTIOC / Nº 7528 ♂; Brasil / R. G. do N / Lab 83 // Coleção Rodolfo Carcavallo // 298 // CTIOC / Nº 7531 ♂; Brasil / R. G. do N / Lab 83 // Coleção Rodolfo Carcavallo // 304 // CTIOC / Nº 7537 ♂; Brasil / R. G. do N / Lab 83 // Coleção Rodolfo Carcavallo // 390 // CTIOC / Nº 7655 ♂; Ceará, Sobral / Sítio Caiçara / Afonso leg // N. 2676 / Hemiptera / Inst. Oswaldo Cruz // CTIOC/ N° 11674 ♀; Ceará, Sobral / Sítio Caiçara / Afonso leg // N. 2677 / Hemiptera / Inst. Oswaldo Cruz //** **CTIOC/ N° 11675 ♂; Sítio Salgadinho / Mun. Guri Branco / Rio Grande Norte / 9-77 // N. 2678 / Hemiptera / Inst. Oswaldo Cruz // CTIOC/ N° 11676 ♀; *Rhodnius nasutus* / Stål, 1859 / 3-947 / H, Lent det // Timbaúba, / (Russas) Ceará / Mangabaira coel. / IV a VI – 1950 // N. 633 / Hemiptera / Inst. Oswaldo Cruz // *Rhodnius brumpti* / Pinto, 1925 ♀ / 7-940 / H. Lent det //CTIOC / N° 11678; *Rhodnius* *nasutus* / Stal, 1859 / 3-947 / H, Lent det // Timbaúba, / Ceará – casa 62 / 14-10-940 / E. Dias leg // N. 635 / Hemiptera / Inst. Oswaldo Cruz // desenhado // *Rhodnius brumpti* / Pinto, 1925 ♀ / 10-940 / H. Lent det //CTIOC / N° 11679; Ilhotas Teresina / Piauí // CTIOC / N° 11682 ♂; *R. nasutus* (?) // CTIOC / N° 11683 ♂; Alencar / Ceará ?? // CTIOC/ N° 11684 ♀.**

*Rhodnius neglectus*

**Non-type material. Brasil / M. Gerais / Lab. 83 // Coleção Rodolfo Carcavallo // 476 // CTIOC / Nº 8317 ♀; Brasil / Minas Gerais / Lab. / v-83 // Coleção Rodolfo Carcavallo // 480 // CTIOC / Nº 8320 ♂; Brasil / Minas Gerais / Lab / VII-85 // Coleção Rodolfo Carcavallo // CTIOC / Nº 8321 ♀; Brasil / Minas Gerais / Lab. 83 // Coleção Rodolfo Carcavallo // 482 // CTIOC / Nº 8322 ♂; Brasil / Minas Gerais / Lab. / v-83 // Coleção Rodolfo Carcavallo // 484 // CTIOC / Nº 8324 ♂; Brasil / M. Gerais / Lab. 83 // Coleção Rodolfo Carcavallo // 486 // CTIOC / Nº 8326 ♀; Brasil / M. Gerais / Lab. 83 // Coleção Rodolfo Carcavallo // 489 // CTIOC / Nº 8329 ♂; Brasil / Minas Gerais / Lab. 83 // Coleção Rodolfo Carcavallo // 491 // CTIOC / Nº 8330 ♀; Brasil / M. Gerais / Lab. 83 // Coleção Rodolfo Carcavallo // 497 // CTIOC / Nº 8332 ♂; Brasil / M. Gerais / Lab. 83 // Coleção Rodolfo Carcavallo // 501 // CTIOC / Nº 8335 ♀; Brasil / S. Paulo / CDVAU 73 // Coleção Rodolfo Carcavallo // 511 // CTIOC / Nº 8344 ♂; Brasil / M. Gerais / Lab. 83 // Coleção Rodolfo Carcavallo // 513 // CTIOC / Nº 8346 ♂; Brasil / M. Gerais / Lab. 83 // Coleção Rodolfo Carcavallo // 515 // CTIOC / Nº 8348 ♂; Brasil / M. Gerais / Lab. 83 // Coleção Rodolfo Carcavallo // 516 // CTIOC / Nº 8349 ♀; Brasil / M. Gerais / Lab. 83 // Coleção Rodolfo Carcavallo // 517 // CTIOC / Nº 8350 ♀; Brasil / M. Gerais / Lab. 83 // Coleção Rodolfo Carcavallo // 525 // CTIOC / Nº 8357 ♀; Itiúba – Bahia / 1969 / Sherlock leg // N. 1958 / Hemiptera / Inst. Oswaldo Cruz // CTIOC / Nº 11953 ♀; Itiúba – Bahia / 1960 / Sherlock leg // N. 1958 / Hemiptera / Inst. Oswaldo Cruz // CTIOC / Nº 11954 ♀; Itiúba – Bahia / 1969 / Sherlock leg // N. 1959 / Hemiptera / Inst. Oswaldo Cruz // CTIOC / Nº 11955 ♀; Itiúba – Bahia / 1969 / Sherlock leg // N. 1961 / Hemiptera / Inst. Oswaldo Cruz // CTIOC / Nº 11956 ♂; Maicurú – Pará / 24.11 – 4.111.1960 / Travassos & Freitas // CTIOC / Nº 11957 ♂; Maicurú – Pará / 24.11 – 4.111.1960 / Travassos & Freitas // CTIOC / Nº 11958 ♂; São Paulo, / C. dos Coqueiros / Freitas leg 6-959 // CTIOC / Nº 11959 ♂; São Paulo, / C. dos Coqueiros / Freitas leg / 6-959 // CTIOC / Nº 11960 ♀; S. Paulo, Cassia / dos coqueiros / Freitas leg. / 6-959 // CTIOC / Nº 11961 ♂; S. Paulo, Cassia / dos coqueiros / Freitas leg. / 6-959 // CTIOC / Nº 11963 ♀; S. Paulo, Cassia / dos coqueiros / Freitas leg. / 6-959 // CTIOC / Nº 11964 ♀; S. Paulo, Emas / Pirassununga / O. Sdiu vant col. / em casa 6-959 // CTIOC / Nº 11965 ♀; Mogi Guaçu / SP – Brasil / IX-68 / J. Mello // CTIOC/ N° 11972 ♂; *Rhodnius neglectus* / Lent, 1954 / à luz / H. Lent Det. // Aragarças – Goiás / 27.xi.56 / j.809 // N. 2741 / Hemiptera / Inst. Oswaldo Cruz // CTIOC Nº 11973 ♀; Faz. De 7 lobos // CTIOC/ Nº 11974 ♀.**
